# Conditional economic incentives and motivational interviewing to improve adolescents’ retention in HIV care and adherence to antiretroviral therapy in Southeast Nigeria: study protocol for a cluster randomised trial

**DOI:** 10.1186/s13063-018-3095-4

**Published:** 2018-12-29

**Authors:** Obinna Ikechukwu Ekwunife, Maureen Ugonwa Anetoh, Stephen Okorafor Kalu, Prince Udegbulam Ele, George Uchenna Eleje

**Affiliations:** 1 Department of Clinical Pharmacy and Pharmacy Management, Nnamdi Azikiwe University, Awka, Nigeria; 2 Virology Laboratory, Nnamdi Azikiwe University Teaching Hospital, Nnewi, Nigeria; 3 Division of Respiratory Medicine, Department of Medicine, Nnamdi Azikiwe University Teaching Hospital, Nnewi, Nigeria; 4 Department of Obstetrics and Gynaecology, Nnamdi Azikiwe University Teaching Hospital, Nnewi, Nigeria

**Keywords:** HIV, AIDS, Adherence, Motivational interviewing, Adolescent, Operational research, Social science, Nigeria

## Abstract

**Background:**

Adolescent HIV patients face enormous difficulty in accessing HIV care services. Given their vulnerability to risk-taking behaviour, this group also have worse treatment outcomes compared to other age groups. Poor treatment outcomes will impact negatively on HIV/AIDS management and control particularly in sub-Saharan Africa (SSA) as more than eight out of ten of the world’s HIV-infected adolescents live in this region of the world. Limited evidence exists on the effectiveness of service delivery interventions to support adolescents’ retention on antiretroviral therapy (ART) and adherence to ART. This trial is designed to evaluate the impact of conditional economic incentive and motivational interviewing on adolescents’ retention in HIV care and adherence to ART in Anambra State, Southeast Nigeria.

**Methods/design:**

The study will be a cluster randomised controlled trial that will be conducted in selected HIV treatment hospitals in Anambra State, Nigeria. Based on sample size calculation, 12 HIV treatment hospitals from Anambra will be selected for the study. Six HIV treatment hospitals each will be randomised to either the intervention or the control arm. A structured adherence support scheme termed the ‘Incentive Scheme’ will be applied to the intervention arm while the control arm will receive routine HIV care (usual care). Additionally, patients in the intervention arm will receive motivational interviewing at baseline and following initiation of antiretroviral therapy (ART), they will receive a gift voucher of US$5.6 when HIV viral load (VL) is < 20 copies/mL at 12 weeks, a gift voucher of US$2.8 if the VL remains suppressed for the next 3 months, and the next 6 months, and finally a gift voucher of US$5.6 if the VL remains < 20 copies/mL at 1 year. All gift vouchers will be conditional not only on VL results but attending the motivational interviews. The primary outcome for the trial will be the difference between groups in the proportion with HIV VL suppression (≤ 20 copies/mL) by 12 months and then 24 months after withdrawal of incentive.

**Discussion:**

The findings of this proposed trial will provide evidence on the feasibility of applying conditional economic incentives combined with motivational interviewing to improve retention and adherence to antiretroviral therapy of adolescents living with HIV in Nigeria and possibly in other sub-Saharan African countries.

**Trial registration:**

Registered in the Pan African Clinical Trials Registry, ID: PACTR201806003040425. Registered on 2 February 2018.

**Electronic supplementary material:**

The online version of this article (10.1186/s13063-018-3095-4) contains supplementary material, which is available to authorized users.

## Background

Adolescent HIV patients face enormous difficulty in accessing HIV care services and they have worse treatment outcomes compared to other age groups [1]. Few studies have investigated HIV-infected adolescents’ progression through the HIV care pathway and have generally found their outcomes to be poor and worse than those for adults or young children. Specifically, rates of retention in care prior to antiretroviral therapy (ART) initiation, as well as retention and adherence to treatment after ART initiation, have been shown to be poor [2, 3]. Furthermore, a systematic review showed an average of only 62% of 12- to 24-year-olds achieved 95% or greater adherence to ART [4]. Poor retention in care and adherence to ART will impact negatively on HIV/AIDS management and control, particularly in SSA as more than eight out of ten of the world’s HIV-infected adolescents live in this region of the world [5].

Poor adherence in adolescents may be connected to the unique psychological, social and health needs that are particular to the adolescent stage. Adolescence is sometimes accompanied by a desire for self-discovery, an emerging sense of autonomy, separation from caregivers and the assertion of independence, the quest for recognition and acceptance, all of which could lead to risk-taking behaviour [6, 7]. Evolving interventions to support retention of HIV-infected adolescents in care as well as ensuring their adherence to ART is essential not only to improve individual health for persons living with HIV but also to reduce transmission.

There is limited evidence on the effectiveness of service delivery interventions to support adolescents’ linkage from HIV diagnosis to ART initiation, retention on ART and adherence to ART. A systematic review by Kim et al. identified a small number of studies that assessed the effectiveness of interventions to support adolescents’ linkage from HIV diagnosis to ART initiation, retention on ART and adherence to ART [4]. The systematic review concluded that offering individual and group education and counselling, financial incentives, increasing clinic accessibility and provision of specific adolescent-tailored services appear promising interventions and warrant further investigation.

### The theoretical basis for conditional economic incentive and motivational interviewing (MI)

There is a theoretical foundation potentially underlying conditional economic incentives (CEI) for HIV treatment adherence. Micro- and behavioural-economic framework suggest that non-adherence is attributed to the perception of reduced utility due to out-of-pocket costs, side effects, perceived risks, delayed benefits, or other idiosyncratic reason [8]. CEI, therefore, increases income when adherent, which in turn increases utility. Additionally, while people know that non-adherence to ART may risk treatment failure, opportunistic infections, etc., these events may occur in the future. Hence, CEI helps to bring the benefits (in monetary terms) much closer to the present.

In Nigeria, the conditional economic incentive has been applied in the Subsidy Reinvestment and Empowerment Programme (SURE-P) Maternal and Child Health (MCH) programme with the aim to increase demand and access to maternal and neonatal health services. In the programme, conditional cash transfer was used to encourage pregnant women from poor households to seek maternal and child health in public hospitals. Conditional cash transfer in a MCH context showed the capacity of having a significant effect on service uptake although the authors concluded that there is a need to track beneficiaries throughout the continuum of care [9].

If appropriately implemented, CEI could help improve patients’ adherence to HIV treatment in the short-term, while the incentives are in place. However, this does not necessarily create a lasting effect after the incentives are removed [8]. Social and behavioural theoretical perspectives explain how behaviour can become habitual and sustained [8]. For instance, the social context that supports satisfaction of basic needs support growth processes, including internally motivated behaviours [10–12]. Additionally, self-determination theory postulates that internalised motivation, rather than external incentives, is essential to long-term behavioural regulation. Therefore, interventions that help self-determination, such as MI, should be coupled to CEI in order to achieve a long-term adherence.

Motivational interviewing is a counselling method informed by self-determination theory and represents a general and practical approach for changing behaviours by enhancing and facilitating a patient’s own internally motivated change process [13]. MI recognises that responsibility for changing behaviour is assumed to lie within the individual, and ambivalence is recognised as a natural part of this change process [8]. MI is different from other adherence counselling technique currently applied in HIV care due to three key elements: MI fosters collaboration between the counsellor and the client; MI evokes or draws out the client’s ideas about change, and MI emphasises the autonomy of the client.

### Study rationale

Based on the systematic review by Kim et al. only one study has assessed the impact of financial incentive coupled with MI on treatment adherence of young adults [14]. The study was conducted using a single-site, non-randomised and applied pre- and post-test design which is not deemed to have high internal validity. Some of the established threats to the internal validity of quasi-experimental design such as pre- and post-test design include history threat, maturation threat, testing threat, lack of blinding, instrumentation, regression to the mean, selection bias and drop out [15].

## Methods/design

### Study objectives

The main aim of the present study is to use the rigour of a randomised controlled trial (RCT) to evaluate the impact of financial incentive and MI on adolescents’ linkage, retention and adherence to antiretroviral therapy and HIV care in Anambra State, Nigeria. A cost-effectiveness analysis alongside the cluster randomised trial will be conducted as a secondary aim to assess the cost per additional patient achieving viral suppression through the proposed intervention. In-depth interview (IDI) with all the healthcare providers in the intervention arm will be conducted at the end of the study to assess their perspective on the programme’s feasibility. The main outcome will be measured at the level of an individual adolescent. Cluster randomisation is being chosen for practical reasons and to prevent treatment group contamination.

### Trial design

**Fig. 1:**
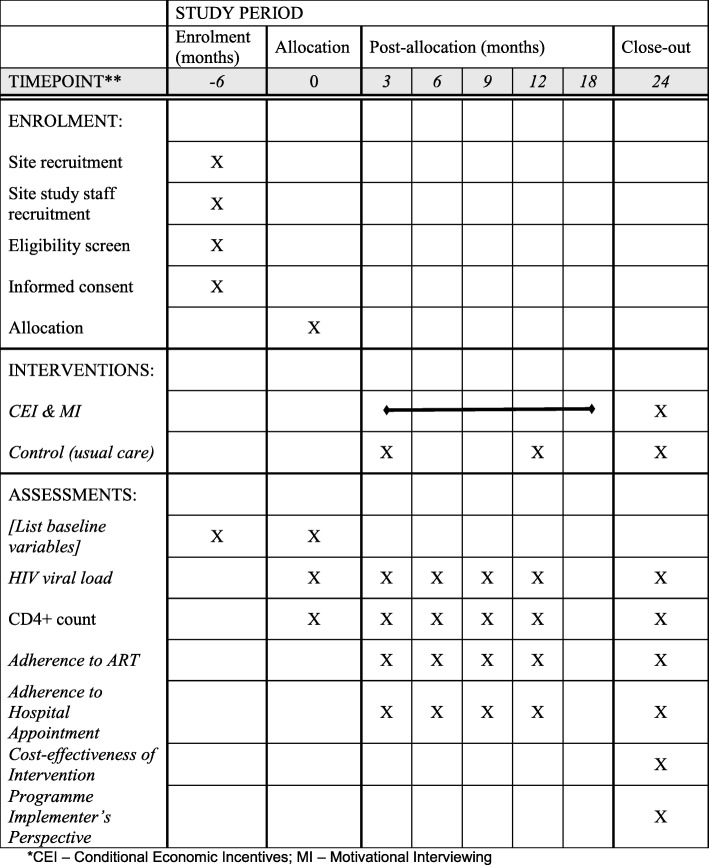
Schedule of enrolment, interventions and assessments. *CEI – Conditional economic Incentives, MI – Motivational interviewing

The study will be a cluster randomised controlled trial. Clusters are HIV treatment hospitals in Anambra state. Hospitals (the clusters) will be matched by type of hospital (e.g. secondary or tertiary) and the paired units will be randomly assigned to the intervention or the control arm. The trial has two periods – intervention period and post-intervention period. Each of these periods will run for a 1-year duration. Further details of the trial are shown in Tables 1 and 2 and Additional file 1. Figure 1 shows the schedule of enrolment, interventions and assessments for the trial design.

**Table 1:** The World Health Organisation (WHO) trial registration dataset

Data category	Information
Primary registry and trial identifying number	The Pan African Clinical Trials Registry: https://pactr.samrc.ac.za/ (PACTR201806003040425)
Date of registration in primary registry	2 February 2018
Source of monetary or material support	European Developing Countries Clinical Trial Partnership (EDCTP)
Primary sponsor	Nnamdi Azikiwe University
Contact for public queries	oi.ekwunife@unizik.edu.ng
Public title	Conditional Economic Incentive and Motivational Interviewing to Improve Adolescents’ Retention and Adherence to Antiretroviral Therapy and Care in South East Nigeria: a Cluster Randomised Trial
Scientific title	Conditional Economic Incentive and Motivational Interviewing to Improve Adolescents’ Retention and Adherence to Antiretroviral Therapy and HIV Care in South East Nigeria: a Cluster Randomised Trial
Country of recruitment	Nigeria
Health condition or problem studies	HIV
Interventions	Conditional economic incentive and motivation interviewing
Key inclusion criteria	All patients with HIV who had transitioned from pediatric stage to a young person/adult; age 10–19 years irrespective of CD4+ cell count; currently off antiretroviral therapy (ART) despite multiple attempts to restart; willing to restart therapy and sign a patient agreement
Study type	Interventional
Date of first enrolment	Yet to commence
Target sample size	240
Recruitment status	Yet to commence
Primary outcome	HIV viral load
Key secondary outcome	CD4+ count, adherence to ART (measured using pill count) and adherence to appointment with hospital

**Table 2:** Protocol for conditional economic incentives

Started ART	VL response and attended for MI	Voucher value (USD)
Week 12	VL < 20	5.6
6 months suppressed	Sustained VL < 20	2.8
9 months suppressed	Sustained VL < 20	2.8
12 months suppressed	Sustained VL < 20	5.6
Total	VL suppression for 12 months	16.8 *ART* antiretroviral therapy, *VL* viral load

### Trial sites selection

Twelve hospitals in Anambra State that offer HIV services will be randomly selected from all the hospitals in the state that provide HIV services. The hospitals included are registered by the National Agency for the Control of AIDS (NACA) to offer HIV services. The hospitals (the clusters) will be matched by type of hospital (e.g. secondary or tertiary) and the paired units will be randomly assigned to intervention or control.

### Participants

Eligibility criteria for participants will include the following: all patients with HIV who had transitioned from pediatric stage to a young person/adult; 10–19 years irrespective of CD4+ cell count; initiated into HIV care and antiretroviral therapy for minimum of 6 months; currently off ART despite multiple attempts to restart; poor medication adherence (< 95% adherence rate assessed through pill count or self-report); willing to restart therapy and sign a written informed consent (see Additional file 2).

### Intervention

A structured adherence support scheme termed the ‘Incentive Scheme’ will be applied to the intervention hospitals while the control hospitals will receive usual care. The Incentive Scheme added to the usual care involves financial incentives linked to viral load (VL) combined with attendance for motivational interviewing (MI) with an adherence counsellor trained in MI techniques. (Table 2) [14]. Training on MI will be in accordance with an MI guide that covers key points for MI as well as examples and key insights to assessing patient risk, risk reduction counselling and inspiring behaviour change [16]. Participants in hospitals randomised to usual care will receive the normal care obtainable in the HIV treatment hospitals. This typically involves monthly or bi-monthly scheduled hospital visit for medical examination, prescription refill and adherence counselling, bi-yearly VL assessment, and yearly CD4^+^ count analysis.

In addition to the usual care, participants in the intervention hospitals will receive MI at baseline and following initiation of ART, they will receive a gift voucher of US$5.6 when VL < 20 copies/mL at 12 weeks, a gift voucher of US$2.8 if the VL remains suppressed for the next 3 months, and the next 6 months, and finally a gift voucher of US$5.6 if the VL remains < 50 copies/mL at 1 year. All incentives will be conditional not only on patients meeting their VL target but attending for MI. Those with VL higher than the thresholds will not receive the incentive. The potential maximum cumulative financial incentive is US$16.8 if viral suppression is sustained for 12 months. The patient will have the liberty to withdraw from the scheme at any time point. Routine hospital data will be collected and outcomes will be assessed by changes in HIV VL and CD4+ count from baseline, at 12 months from enrolment and at 24 months to establish whether any benefit is seen from the intervention could be sustained when financial incentives cease. Each patient in the study will be assigned to a study nurse working in the HIV treatment hospital and they will be tracked with their mobile phone numbers.

### Outcomes

The primary outcome for the trial will be the difference between groups in the proportion with HIV VL suppression (≤ 20 copies/mL) by 12 months and 24 months. The secondary outcome measure will include the average change in CD4+ count, adherence to ART (measured using pill count) and adherence to an appointment with the hospital for individual participants. Viral load analysis will be done using the COBAS TaqMan 96 and the Amplilink software.

### Laboratory methods

Ten millilitres of whole blood will be collected from each patient into a Vacutainer EDTA-plasma specimen from patients who were confirmed to be HIV-1 seropositive and receiving ART. These EDTA-plasma samples will be submitted to the virology laboratory of Nnamdi Azikiwe University Teaching Hospital for routine HIV-1 VL testing by the CAP/CTM v2.0 (Switzerland, 2016). EDTA-blood tubes will be centrifuged at 1450 *g* for 25 min prior to the plasma being separated into two aliquots in 2-mL cryovials and stored at − 88 °C in an ultralow freezer until the time of testing.

The VLs of these samples will be measured using the Roche COBAS AmpliPrep/COBAS TaqMan (CAP/CTM) commercial kits according to the manufacturer’s instructions. The plasma HIV-1 ribonucleic acid (RNA) levels will be determined as copies/mL.

Viral RNA will be extracted automatically and transferred to the COBAS TaqMan system for amplification and detection. The CAP/CTM v2.0 will be implemented along with the COBAS AmpliPrep automated nucleic acid extractor and TaqMan 96 analyser. The quantification range of the CAP/CTM v2.0 is from 20 copies/mL to 10^7^ copies/mL. Each run will consist of tested samples and one high-positive control, one low-positive control, and one negative control from the kits. Results will be validated only when the three controls (NC, LPC and HPC) have passed per run. The CD4+ count analysis will be performed using the CD4+ easy count kit and the Partec CyFlow (Görlitz, Germany, 2014) in accordance with the manufacturer’s instructions.

### Sample size

The sample size was calculated using a web-based sample size calculator of the UCSF Clinical and Translational Science Institute [17]. The sample size calculation is based on the proportion of patients with VL suppression ≤ 20 copies/mL – the main outcome measure. Based on a power of 80% and an α of 0.05 (two-sided), 63 patients per group will be needed to observe a 12% (assumed standard deviation of 24%) increase in the number of participants with VL suppression as previously reported [18]. After adjustment for the cluster design, based on the assumed intracluster correlation coefficient of 0.047 and a fixed cluster per arm of six HIV treatment hospitals, the effective sample size increased to 120 patients per arm (i.e. a total of 240 participants). Due to potential attrition that could arise as a result of severe adverse events, treatment failures or the participant simply deciding to withdraw, we added three participants per hospital increasing the number of participants in each arm to 138 (i.e. a total of 276 participants). A fixed number of six clusters or hospitals per arm (i.e. 12 hospitals in total for the trial) were informed by the fact that only 12 hospitals in Anambra State offer comprehensive HIV services including HIV-adherence counselling and antiretroviral treatment services and these 12 hospitals have appreciable HIV client load. Each of the hospitals will, therefore recruit 23 participants.

### Randomisation

We will stratify each cluster unit (HIV treatment hospital) according to the type of clinic (secondary or tertiary). We will randomly allocate each HIV treatment hospital in each stratum into the intervention arm or the control arm. The randomisation schedule will be done using Research Randomiser, a web-based computer random-number generator [19]. The randomisation schedule will be carried out by an independent person who is not part of the research team.

### Statistical analysis

Statistical analysis will be conducted using IBM SPSS for Windows, Version 20 (IBM Corp, Armonk, NY, USA). As shown in Table 3, descriptive statistics will be used to evaluate differences in demographic and clinical characteristics. Categorical variables will be expressed as frequencies or percentages and quantitative variables as means and standard deviations or medians and interquartile ranges. An intention-to-treat analysis will be conducted. A secondary per-protocol analysis will also be conducted. The latter will include only those participants who completed the treatment protocol originally allocated, providing results on the efficacy of the trial. If there is a significant difference between the intention-to-treat analysis and the per-protocol analysis, then the former will be reported. Comparison of dichotomous measurements between the intervention group and the control group will be conducted using the chi-square test (or Fisher’s exact test) while comparison of the mean of continuous measurements between the intervention group and the control group will be conducted using an independent *t* test. A two-sided *p* value of 0.05 will be used to indicate statistical significance.

**Table 3:** Data analysis outline

Study objective	Outcome variable	Type of analysis
Objective 1: effectiveness of intervention	Primary outcome	
	HIV viral load	*χ*^2^ test or Fisher’s exact test
	Secondary outcomes	
	CD4+ count	Within group: paired-sample *t* tests or Wilcoxon signed-rank tests; between group – Student’s *t* test or Wilcoxon signed-rank tests
	Adherence to antiretroviral therapy (ART)	*χ*^2^ test or Fisher’s exact test
	Adherence to hospital appointment	Within group: paired-sample *t* tests or Wilcoxon signed-rank tests; between group – Student’s *t* test or Wilcoxon signed-rank tests
Objective 2: cost-effectiveness analysis	Cost-effectiveness of intervention	Incremental cost-effectiveness ratio
Objective 3: qualitative assessment of implementer’s perspective	Programme implementer’s perspective	In-depth interview (thematic content analysis)

Health resource implication and cost-effectiveness of the Incentive Scheme intervention will be assessed. Health resource use will be estimated from the health provider’s perspective, i.e. all cost incurred for providing the Incentive Scheme by a health service provider. Resource use items will include items such as staff time, consumables, cost of financial incentive and equipment. All patient and family resources will be excluded. An activity-based costing method will be used to measure resource use. Data-capture questionnaires will be developed and sent to all the study personnel in the different study sites to record all their activities or resource use. Resource use items will then be multiplied by unit costs to determine a cost item. The entire cost item will be summed up and divided by the number of participants to determine the cost of the incentive scheme intervention per patient. A cost-effectiveness analysis alongside the cluster randomised trial will be conducted to assess the cost per additional patient achieving viral suppression through the proposed intervention. Incremental cost-effectiveness ratio (ICER) of Incentive Scheme over usual care will be calculated to determine the cost-effectiveness of the former.

A in-depth interview (IDI) with all the health providers in the intervention arm will be conducted at the end of the study to assess their perspective on the programme’s feasibility. The IDI instrument to be used will be developed using the Pathfinder International Tool Series guideline on conducting IDI [20]. During the interview, notes and audio digital recording will be taken and non-verbal and gestural cues will also be observed. All recorded data will be transcribed verbatim into English by the researcher and research assistant independently. A thematic content approach, guided by the Graneheim and Lundman framework, will be utilised for qualitative data [21]. Responses from the IDIs will be read systematically through in order to indentify the meaning units. A meaning unit will be defined as a string of the text that expressed a single coherent thought, up to the point that the coherent thought changed [22]. The meaning units will be coded using a describing cue related to the content of the meaning unit. Codes concerning the same subject will be grouped together into categories. The interview guide will be used as a point of departure for grouping information, deductively. Information obtained during the IDIs will be analysed and merged according to the codes and themes. Original data will be reassessed after analysis in order to detect any concepts or information that may be missed.

### Recruitment and retention

Eligible adolescents will be recruited by the study personnel in the trial site (e.g. trial physician or nurse). Each study participant will be assigned to a study nurse working in the HIV treatment hospital and they will be tracked with their mobile phone numbers.

### Trial subject safety

The major risk envisaged in this trial will be exposure of the data of study participants. In order to mitigate this risk, patients’ data will be treated with confidentiality. All personal data collection and processing will be carried out according to European Union (EU) and national legislation. Personal data collected are those necessary to establish the primary and secondary study outcomes. Unique identifiers and a password-protected database will be used to protect the personal information of the study participant. We will keep a written document with detailed information of the origin of all used human samples. All the study partners in the different study sites will receive training on procedures for handling pseudonymised data during study briefing/training. Training will be based on the principle of handling personal data elucidated in the ‘Article 29 Data Protection Working Party’. To ensure the protection of personal data, the principal investigator (PI) (including data analyst) will receive only key-coded data.

### Management of incidental findings and data monitoring

There is a chance of anticipatable incidental findings due to the increased number of laboratory testing (especially for those in the intervention arm) and closer monitoring. These anticipatable incidental findings include early detection of the following:

1. Clinical failure: this is the presence of new or recurrent clinical event indicating severe immunodeficiency (WHO clinical stage 4 conditions) following 6 months of effective treatment
2. Immunological failure: this represents a CD4+ cell count fall to or below pre-treatment baseline value or persistent CD4+ levels below 100 cells/mm^3^ or 50% decline from on-therapy CD4+ cell count peak level
3. Virologic failure: this is the persistently detectable VL exceeding 1000 copies/mL (that is, two consecutive VL measurements within a 3-month interval, with adherence support between measurements) after at least 6 months of using antiretroviral drugs

Any such anticipatable incidental findings will be disclosed to the participants and their parents/legal guardians (for those less than 18 years). Management of these anticipatable incidental findings will be in accordance to the National Guidelines for HIV Prevention, Treatment and Care. This entails the following: providing adherence support and treating opportunistic infections in any suspected case of treatment failure; reassessing VL and.

CD4+ improvement after 3 months; and changing to second-line therapy in case of no improvement (i.e. VL > 1000 copies/mL).

Any study-related adverse events (AEs) will be documented and reported to the Steering Committee/Data and Safety Monitoring Board. In the case of a serious adverse event (SAE), it will be reported to the Nnamdi Azikiwe University Ethics Committee within 14 days of the PI becoming aware of the event. SAE is defined as an untoward occurrence that (1) results in death; (2) is life-threatening; (3) requires hospitalisation or prolongation of existing hospitalisation; (4) results in persistent or significant disability or incapacity; (5) consists of a congenital abnormality or birth defect; and (6) is otherwise considered medically significant by the investigator.

Conditions in which expedited reporting will not apply include hospitalisation for (1) treatment which was pre-planned, or for a pre-existing condition not associated with any deterioration in condition and (2) treatment on an emergency, outpatient basis for an event not fulfilling any of the definitions of serious as given above and not resulting in hospital admission. Patients will be asked if any adverse events have occurred when they attend for any trial-related visits.

### Trial management

The Steering Committee will also serve as the Data and Safety Monitoring Board (DSMB). This committee is composed of two of the authors and three external, independent experts in HIV care, pharmacology and pharmacy (Appendix). The Steering Committee/DSMB will provide overall supervision of the trial. Specifically, the committee will: (1) review and evaluate the accumulated study data every 6 months for patient safety and (2) study the conduct and progress of the trial and make appropriate recommendation to the trial team.

### Trial monitoring

A standard operating procedure defining the responsibilities, processes and deadlines for the monitoring of the ARA trial as well as a monitoring manual will be prepared. This is to ensure uniform monitoring of all the sites in the trial.

### Clinical trial registration

This trial is registered in the WHO International Clinical Trials Registry through the WHO International Registry Network (https://pactr.samrc.ac.za/: PACTR201806003040425).

## Discussion

The findings of this proposed trial should provide evidence on the feasibility of applying conditional economic incentives combined with MI to improve retention and adherence to antiretroviral therapy of adolescents living with HIV in Nigeria and possibly in other sub-Saharan African countries.

We anticipate that enrolment of adolescents with HIV might be slow especially in HIV treatment hospitals with a low patient load. However, we included 12 HIV treatment hospitals that offer comprehensive HIV counselling and antiretroviral treatment services with good client load (including adolescents).

## Trial status

This is protocol version 1.0 as at 23 August 2018. Recruitment is planned to begin on 1 December 2018 and completed on 28 February 2019.

### Additional files

Additional file 1: Standard Protocol Items: Recommendations for Interventional Trials (SPIRIT) Checklist. (DOCX 20 kb)

Additional file 2: Informed consent form (DOC 53 kb)

## Appendix

The following person are members of the Trial Steering Committee/Data and Safety Monitoring Board (DSMB):

**Table 4:** Members of Steering Committee/DSMB

Name	Job description	Institution
Prof. PU Ele	Professor of Internal Medicine; HIV focal person, NAUTH	Nnamdi Azikiwe University Teaching Hospital (NAUTH)
Prof. OJ Afonne	Professor of Pharmacology	Nnamdi Azikiwe University Teaching Hospital (NAUTH)
Dr. Raymond Okechukwu	Pharmacist with specialisation in HIV care	Nnamdi Azikiwe University Teaching Hospital (NAUTH)
Prof. CO Esimone	Professor of Pharmaceutical Microbiology and Biotechnology, Deputy Vice Chancellor or Nnamdi Azikiwe University	Nnamdi Azikiwe University (NAU)
Dr. GU Eleje	Consultant in Obstetrics and Gynaecology	Nnamdi Azikiwe University Teaching Hospital (NAUTH)

## Abbreviations

AIDS: Acquired immunodeficiency syndrome
ART: Antiretroviral therapy
CEI: Conditional economic incentive
HIV: Human immunodeficiency virus
ICER: Incremental cost-effectiveness ratio
IDI: In-depth interview`
MCH: Maternal and child health
MI: Motivational interviewing
NACA: National Agency for the Control of AIDS
NGN: Nigerian Naira
RCT: Randomised controlled trial
SPSS: Statistical Package for the Social Sciences
SSA: Sub-Saharan Africa
SURE-P: The Subsidy Reinvestment and Empowerment Programme
UCSF: The University of California, San Francisco
VL: Viral load
WHO: World Health Organisation

## References

[1] MacPherson P, Munthali C, Ferguson J, Armstrong A, Kranzer K, Ferrand RA (2015). Service delivery interventions to improve adolescents’ linkage, retention and adherence to antiretroviral therapy and HIV care. Tropical Med Int Health.

[2] Fox MP, Rosen S (2015). Systematic review of retention of pediatric patients on HIV treatment in low and middle-income countries 2008–2013. AIDS (London, England).

[3] Philbin MM, Tanner AE, DuVal A, Ellen JM, Xu J, Kapogiannis B (2014). Factors affecting linkage to care and engagement in care for newly diagnosed HIV-positive adolescents within fifteen adolescent medicine clinics in the United States. AIDS Behav.

[4] Kim SH, Gerver SM, Fidler S, Ward H (2014). Adherence to antiretroviral therapy in adolescents living with HIV: systematic review and meta-analysis. AIDS (London, England).

[5] Adejumo OA, Malee KM, Ryscavage P, Hunter SJ, Taiwo BO (2015). Contemporary issues on the epidemiology and antiretroviral adherence of HIV-infected adolescents in sub-Saharan Africa: a narrative review. J Int AIDS Soc.

[6] Chein J, Albert D, O’Brien L, Uckert K, Steinberg L (2011). Peers increase adolescent risk taking by enhancing activity in the brain’s reward circuitry. Dev Sci.

[7] Gardner M, Steinberg L (2005). Peer influence on risk taking, risk preference, and risky decision making in adolescence and adulthood: an experimental study. Dev Psychol.

[8] Galarraga O, Genberg BL, Martin RA, Barton Laws M, Wilson IB (2013). Conditional economic incentives to improve HIV treatment adherence: literature review and theoretical considerations. AIDS Behav.

[9] Okoli U, Morris L, Oshin A, Pate MA, Aigbe C, Muhammad A (2014). Conditional cash transfer schemes in Nigeria: potential gains for maternal and child health service uptake in a national pilot programme. BMC Pregnancy Childbirth.

[10] Deci EL, Koestner R, Ryan RM (1999). A meta-analytic review of experiments examining the effects of extrinsic rewards on intrinsic motivation. Psychol Bull.

[11] Deci EL, Ryan RM (1990). A motivational approach to self: integration in personality. Nebr Symp Motiv.

[12] Ryan RM, Deci EL (2000). Intrinsic and extrinsic motivations: classic definitions and new directions. Contemp Educ Psychol.

[13] Miller WR, Rose GS (2009). Toward a theory of motivational interviewing. Am Psychologist.

[14] Foster C, McDonald S, Frize G, Ayers S, Fidler S (2014). ‘Payment by Results’—financial incentives and motivational interviewing, adherence interventions in young adults with perinatally acquired HIV-1 infection: a pilot program. AIDS Patient Care STDs.

[15] Krass I (2016). Quasi experimental designs in pharmacist intervention research. Int J Clin Pharm.

[16] Cook PF, Corwin MA, Bradley-Springer L (2013). Motivational interviewing and HIV: reducing risk, inspiring change.

[17] UCSF. Sample Size Calculators. UCSF. 2016. http://www.sample-size.net/means-sample-sizeclustered/. Accessed 9 December 2016.

[18] Farber S, Tate J, Frank C, Ardito D, Kozal M, Justice AC (2013). A study of financial incentives to reduce plasma HIV RNA among patients in care. AIDS Behav.

[19] Urbaniak GC, Plous S. Research Randomizer Version 4.0 ed 2013. https://www.randomizer.org/. Accessed 10 Dec 2016.

[20] Boyce C, Neale P. Conducting In-depth interviews: a guide for designing and conducting in-depth interviews for evaluating input. Pathfinder International 2006. http://www2.pathfinder.org/site/DocServer/m_e_tool_series_indepth_interviews.pdf. Accessed 6 Dec 2016.

[21] Graneheim UH, Lundman B (2004). Qualitative content analysis in nursing research: concepts, procedures and measures to achieve trustworthiness. Nurse Educ Today.

[22] Falnes EF, Moland KM, Tylleskar T, de Paoli MM, Msuya SE, Engebretsen IM (2011). ‘It is her responsibility’: partner involvement in prevention of mother to child transmission of HIV programmes, northern Tanzania. J Int AIDS Soc.

[23] The Helsinki Declaration of the World Medical Association (WMA). Ethical principles of medical research involving human subjects. Pol Merkur Lekarski. 2014;36(215):298–301. 24964504

