# Supplementary material for: Conditional economic incentives and motivational interviewing to improve adolescents’ retention in HIV care and adherence to antiretroviral therapy in Southeast Nigeria: study protocol for a cluster randomised trial
Source: Trials. 2018 Dec 29;19:710. doi: 10.1186/s13063-018-3095-4 (PMC6311063; doi:10.1186/s13063-018-3095-4)
Supplement: Supplementary file 2 — Informed consent form (DOC 53 kb) [file 13063_2018_3095_MOESM2_ESM.doc]

# Additional file 2: Informed consent form

*Informed Consent Form for Participants*

IRB Research approval number: NAUTH/CS/66/VOL.11/092/2018/052

**Title of the research:** Conditional economic incentives and motivational interviewing to improve adolescents’ retention and adherence to antiretroviral therapy and HIV care in Nigeria: a cluster randomized trial

**Name and affiliation of the researcher or applicant:** Dr. Obinna Ekwunife of Nnamdi Azikiwe University

**Sponsor(s) of research:** Nnamdi Azikiwe University, Along Enugu-Onitsha Expressway Ifite Awka NG, 420110

**Purpose(s) of research:** The purpose of this research is to find out whether financial incentive coupled with motivational interviewing could lead to better retention of adolescents in HIV care and increase their adherence to antiretroviral therapy.

**The procedure of the research, what shall be required of each participant and approximate total number of participants that would be involved in the research:** We will randomize the hospitals in this study into two groups. One group will receive conditional financial incentive coupled with motivational interviewing and the other will receive the usual care. You will be required to visit your hospital once a month as usual. The financial incentive ranges from NGN 1000 – NGN 2000. Motivational interviewing is a chat with a nurse for about 10 minutes. Your child will be required to visit the hospital once a month as usual. In total, we expect to recruit 20 clinics and 240 participants into this study throughout Anambra state.

**Expected duration of research and of the participant(s)’ involvement:** In total, we expect you to be involved in this research for two years. You should not spend more than 1 hour extra at each clinic visit.

**Risk(s):** We do not foresee any risk that could arise from this study.

**Costs to the participants, if any, of joining the research:** Your participation in this research will not cost you anything.

**Benefit(s):** The goal of this research is to find ways to increase your retention in care and adherence to therapy which will translate to sustained viral load suppression, improved and prolonged quality of life. We hope that our proposed intervention which is the conditional financial incentive coupled with motivational interviewing (incentive scheme) will be able to achieve this. Additionally, you will receive proper monitoring and prompt treatment.

**Confidentiality:** All information and blood samples collected in this study will be given code numbers and no name will be recorded. This cannot be linked to you in anyway and your name or an identifier will not be used in any publication or reports from this study.

**Voluntariness:** Your participation in this research is entirely free.

**Alternatives to participation:** If you choose not to participate, this will not affect your treatment in this hospital in any way.

**Due inducement(s):** You will not be paid any fees for participating in this research.

**Consequences of participants’ decision to withdraw from research and procedure for orderly termination of participation:** You can also choose to withdraw from the research at any time. Please note that some of the information that has been obtained from you before you choose to withdraw may have been used in reports and publications. These cannot be removed anymore. Such information will however, never be traceable to you as a person. The researchers promise to comply with your wishes as much as is practicable.

**The modality of providing treatments and action(s) to be taken in case of injury or adverse event(s):** If you suffer any injury as a result of your participation in this research, you will be treated at the Nnamdi Azikiwe University Teaching Hospital and the research will bear the cost of this treatment.

**What happens to research participants and communities when the research is over:** The researchers will inform you of the outcome of the research through a published scientific paper. During the course of this research, you will be informed of any information that may affect your continued participation or your health.

**The statement about sharing of benefits among researchers and whether this includes or exclude research participants:** There is no benefit foreseen to be shared from this research other than that the result may be applied to help improve retention in care and increase adherence to antiretroviral therapy.

**Any apparent or potential conflict of interest:** None of the researchers have the potential conflict of interest. We are not aware of any other information that may cause the researchers not to do their work with fear or favour.

**Statement of person obtaining informed consent:**

I have fully explained this research to ____________________________________ and have given sufficient information, including about risks and benefits, to make an informed decision.

DATE: _____________________ SIGNATURE: _______________________________

NAME: ______________________________________________

**Statement of person giving consent:**

I have read the description of the research or have had it translated into language I understand. I have also talked it over with the doctor to my satisfaction. I understand that my participation is voluntary. I know enough about the purpose, methods, risks and benefits of the research study to judge that I want to take part in it. I understand that I may freely stop being part of this study at any time.

DATE: ___________________ SIGNATURE: _________________________________

NAME: _____________________________________________

LANGUAGE: _________________________________

WITNESS’ SIGNATURE (if applicable): ___________________________

WITNESS’ NAME (if applicable): ______________________________________

**Detailed contact information including contact address, telephone, fax, e-mail and any other contact information of researcher(s), institutional HREC and head of the institution:** This research has been approved by the Health Research Ethics Committee of the Nnamdi Azikiwe University and the Chairman of this Committee can be contacted at Ethical Clearance Office, Nnamdi Azikiwe University Teaching Hospital, Nnewi. In addition, if you have any question about your participation in this research, you can contact the principal investigator, Dr. Obinna Ekwunife at his office in Department of Clinical Pharmacy & Pharmacy Management, Nnamdi Azikiwe University, Agulu. The phone number is 234(0)7062032501.
